# Isolated single sound lip-reading using a frame-based camera and event-based camera

**DOI:** 10.3389/frai.2022.1070964

**Published:** 2023-01-11

**Authors:** Tatsuya Kanamaru, Taiki Arakane, Takeshi Saitoh

**Affiliations:** Kyushu Institute of Technology, Fukuoka, Japan

**Keywords:** event-based camera, frame-based camera, lip-reading, single sound recognition, face detection, facial feature points detection, Temporal Convolutional Network

## Abstract

Unlike the conventional frame-based camera, the event-based camera detects changes in the brightness value for each pixel over time. This research work on lip-reading as a new application by the event-based camera. This paper proposes an event camera-based lip-reading for isolated single sound recognition. The proposed method consists of imaging from event data, face and facial feature points detection, and recognition using a Temporal Convolutional Network. Furthermore, this paper proposes a method that combines the two modalities of the frame-based camera and an event-based camera. In order to evaluate the proposed method, the utterance scenes of 15 Japanese consonants from 20 speakers were collected using an event-based camera and a video camera and constructed an original dataset. Several experiments were conducted by generating images at multiple frame rates from an event-based camera. As a result, the highest recognition accuracy was obtained in the image of the event-based camera at 60 fps. Moreover, it was confirmed that combining two modalities yields higher recognition accuracy than a single modality.

## 1. Introduction

The typical means of communication for us humans is voice. Audio-based speech recognition (ASR) that analyzes this voice and estimates the contents of the speech has been researched for a long time. Furthermore, our lives have become more comfortable by ASR technology has been put to practical use in smartphone applications. However, there are situations in which ASR is difficult to use, as listed below. (1) People who have difficulty hearing the other person's voice due to hearing impairment, people who cannot speak due to speech problems, and have difficulty communicating their voice. (2) In a noisy environment such as a car, train, factory, or lively place such as festivals. (3) Places where it is difficult to speak out, such as public places. (4) When multiple people speak simultaneously, (5) Restoration of the utterance content only from visual information without audio information. To solve these problems, studies using other modalities are ongoing. Among them, lip-reading or visual speech recognition (VSR) technology uses a camera image and estimates utterance content from visual information as a typical modality. Research on lip-reading technologies has been undertaken since the 1950s (Hao et al., 2020). In recent years, recognition accuracy has been greatly improved by enhancing deep learning (Afouras et al., 2018) and public datasets (Chung and Zisserman, 2016; Chung et al., 2017; Saitoh and Kubokawa, 2018).

However, the sampling frequency in general ASR technology is 16 kHz, while that in lip-reading technology is 30 Hz, and there is a large difference in time resolution. It has been reported that the recognition accuracy is improved by using a high-speed camera, but it is expensive. Therefore, in this research, we focus on an event-based camera, which is inexpensive, has high frame rates, and has low power consumption.

The event-based camera is a sensor inspired by living organisms' retinas and has features such as high time resolution and high dynamic range. Unlike conventional frame-based cameras, event data is saved when it is determined that an event has occurred due to a temporal change in the brightness value for each pixel.

In event-based camera research, many studies generate images similar to frame images from the event data. However, this paper focuses on analyzing faces rather than reconstructing images exactly. A face extraction method has been proposed for event-based as well as frame-based cameras (Ramesh and Yang, 2020). Lenz et al. (2020) have proposed a face-tracking method that utilizes the characteristics of an event-based camera by using blinks. In this study, the detection and tracking of the subject's face are started with a single blink. It also shows robustness regarding the distance between the subject and the camera and the simultaneous detection of multiple people. In addition, this paper studies lip-reading using an event-based camera.

Li et al. (2019) reported lip-reading using an event-based camera. They played back the speech scenes of GRID corpus (Cooke et al., 2006), a publicly available audio-visual benchmark dataset shot with a standard video camera, on a screen and shot the scenes with an event-based camera. In other words, their studies did not directly shoot speech scenes using an event-based camera. The GRID scenes were shot with a frame-based camera, and the time resolution was 25 Hz. Thus, this does not take full advantage of the high time resolution characteristic of the event-based camera. To the best of the author's knowledge, no other lip-reading research directly shot speech scenes with an event-based camera. Therefore, this paper is the world's first lip-reading for data shot directly with an event-based camera.

The recognition targets in the lip-reading field are roughly classified into three categories: single sounds (Nakamura et al., 2021), words (Chung and Zisserman, 2016; Saitoh and Kubokawa, 2018), and sentences (Assael et al., 2016; Chung et al., 2017; Afouras et al., 2018; Shirakata and Saitoh, 2021). In research using frame-based cameras, single sounds were the research target in the early days of lip-reading technology, but it was not easy to identify consonants. In recent years, words and sentences have been actively discussed because words or sentences are more expansive than single sounds. This paper focuses on single sounds that are difficult to recognize in the frame-based camera.

The contributions of our works are as follows.

- We show that face detection and facial feature points detection can be performed on face images taken with an event-based camera.
- We show that lip-reading can be performed from the utterance scene taken with the event-based camera.
- This paper shows that the event-based camera has higher lip-reading accuracy than the traditional frame-based camera.
- We show that combining two modalities, the frame-based camera and the event-based camera, provides higher accuracy than a single modality.

The rest of this paper is organized as follows: Section 2 describes the proposed method. Section 3 describes some experimental results. This paper concludes in Section 4.

## 2. Proposed method

**Figure 1:**
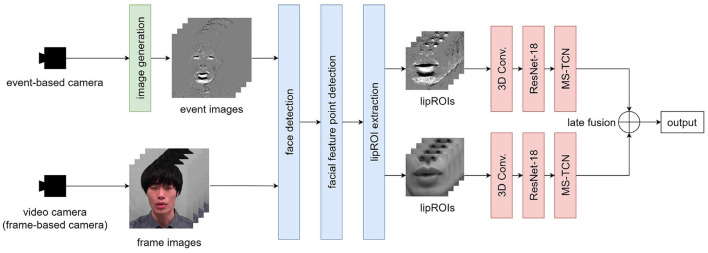
Processing pipeline of the proposed method.

The overview of the proposed method is shown in Figure 1. A speech scene is simultaneously shot using a standard video camera and an event-based camera. Event images are generated by applying the image generation method described in Section 2.1 to the data of the event-based camera. Face detection and facial feature points detection is applied to event and frame images, and lipROIs, a region of interest around the lip shown in Figure 1, is extracted for each. lipROIs are set as input data, and each modality's output is obtained by the recognition model described in Section 2.4. We apply late fusion to get the final output as a final process.

### 2.1. Image generation

When the frame rate is expressed as *F*[fps], the frame-based camera captures *F* images per second. On the other hand, each pixel independently records the event information (change polarity, coordinates, and time) of the brightness change in the event-based camera. Therefore, there is no concept of frame rate in the event data recorded by the event-based camera. Image generation from event data assigns pixel values based on the event data of each pixel within a certain time.

This paper sets a pseudo frame rate *F* for image generation from the event data. We count the number of events at pixel (*x, y*) within the 1/*F*[second] interval from the recording start time. There are two types of events, positive and negative, and the sum of *S*(*x, y*) is calculated with positive as +1 and negative as −1. The following equation gives the pixel value of the image *E* (denoted as an event image) generated by the event-based camera.


$$\begin{array}{l} E\left(x,y\right)=\{\begin{array}{ll} 0 & S\left(x,y\right)<0\\ 128 & S\left(x,y\right)=0\\ 255 & S\left(x,y\right)>0 \end{array} \end{array}$$

**Figure 2:**
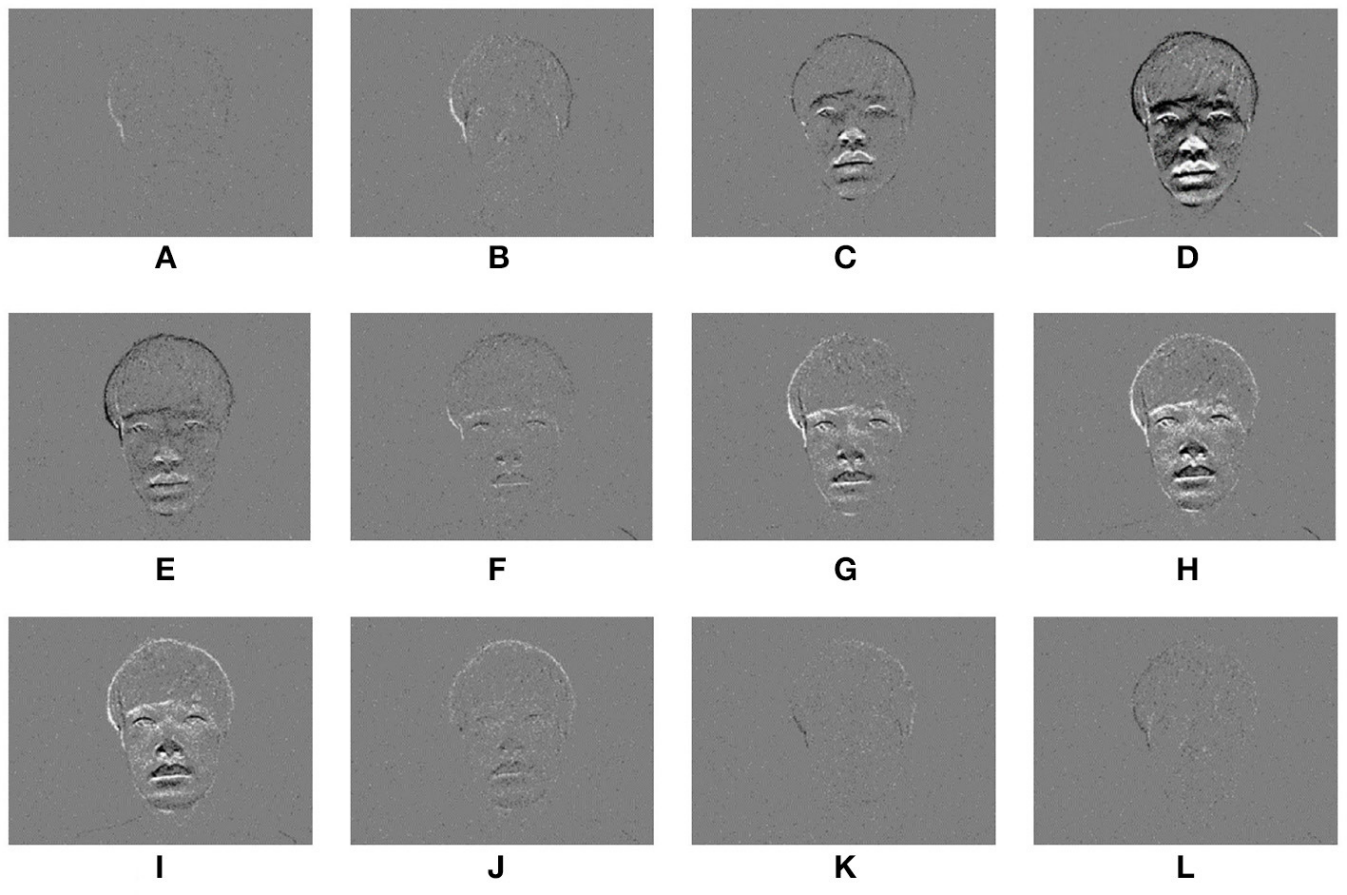
Face images taken with an event-based camera during nodding. **(A–L)** are a time-series event images in one shooting scene.

Figure 2 shows an example of a time-series event image generated from the event data acquired when one person sits on a chair in front of the event-based camera and nods. Figure 2A is the first state of facing the front and resting, Figure 2D is the state of facing down, Figure 2F is the state of returning to the front again, and Figure 2L is the state of facing the front and resting. The gray value is a pixel with no movement in the event image, and the white or black is a pixel with movement. When the luminance value is higher than the previous time, it becomes a white pixel; when the luminance value is lower, it becomes a black pixel. In Figures 2C–E, G–I, the edge of the face can be observed in the event image by the movement of the head.

**Figure 3:**
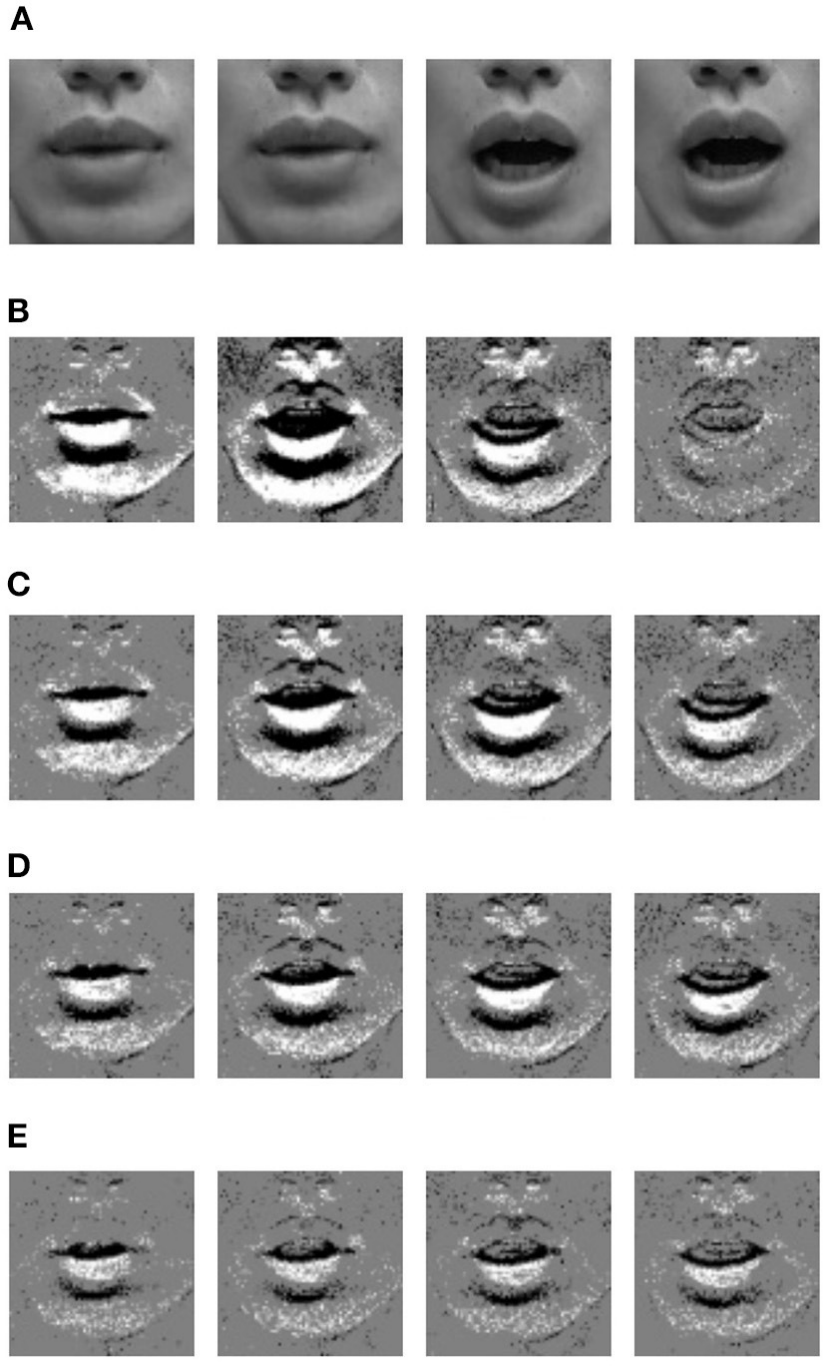
LipROIs of frame images and event images when uttering sound /A/. **(A)** Frame image @29.97 fps. **(B)** Event image @29.97 fps. **(C)** Event image @60 fps. **(D)** Event image @100 fps. **(E)** Event image @200 fps.

Furthermore, by changing *F*, an event image with a higher frame rate than the camera image can be generated. Figure 3 shows the lipROIs described later. In the figure, Figure 3A is a camera image with a frame rate of 29.97 fps, and Figures 3B–E are event images with different frame rates. Four images are shown in Figures 3A–E, but each row is an image taken at the same time. When observing these event images, the number of events decreases as the frame rate increases, so the gray value increases overall, but it is thought that small movements can be captured.

### 2.2. Face detection

Figure 2A is a stationary state before starting the nodding motion. By observing only this image, the exact shape of the face cannot be seen. When shooting a subject with a frame-based camera, if the subject is within the camera's field of view, the subject can be shot even if it is stationary. On the other hand, in the case of an event-based camera, it is impossible to capture a stationary subject, and the subject's data is not saved unless there is a relatively apparent movement due to the subject or camera movement. Therefore, when shooting the utterance scene with the event-based camera, we take the means of having the subject perform the nodding motion first, as shown in Figure 2.

In order to detect facial feature points, it is desirable that edges can be observed in the target image. Since the nodding motion is a reciprocating motion of lowering and raising the head, an image with many black and white pixels can be observed in a series of motions. Therefore, for each image, the average luminance value of all pixels is calculated, and the image with the minimum average luminance value in the nodding motion is automatically extracted as a black image. This paper applies face detection and facial feature points detection to the black image.

Before detecting facial feature points, it is necessary to specify the facial position. Therefore, the face detection process from the event image is applied. Various methods have been proposed for face detection for conventional frame-based cameras. Using Haar-like features and histogram of oriented gradients (HOG) features have been proposed as non-deep learning-based methods (Viola and Jones, 2001; Dalal and Triggs, 2005). As deep learning-based methods, Deep Pyramid Deformable Parts Model for Face Detection (DP2MFD) (Ranjan et al., 2015), Deep Dense Face Detector (DDFD) (Farfade et al., 2015), and Retinaface (Deng et al., 2020) have been proposed. In this research, the object detection method using the HOG feature proposed by Dalal and Triggs, which is proposed in the frame-based camera and implemented in dlib^1^ (Dalal and Triggs, 2005) is applied to the event image. In preliminary experiments, we applied dlib, MediaPipe Face Detection^2^, and RetinaFace (Deng et al., 2020), and dlib had the highest accuracy.

### 2.3. Facial feature points detection

**Figure 4:**
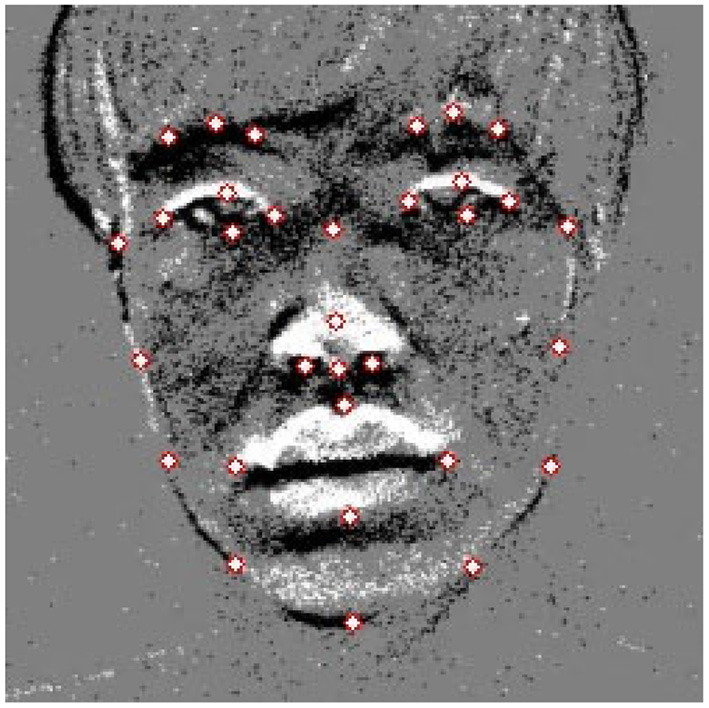
Facial feature points (face contour: 9 points, eyebrows: 3 points, eye contour: 4 points, nose: 5 points, lip: 4 points).

Facial feature points have semantic meaning, also known as facial landmarks or fiducial points. Facial feature points are mainly located around facial components such as the eyes, mouth, nose, and chin. A typical facial feature points detection method for frame-based cameras is the method of Kazemi and Sullivan (2014) implemented in dlib, and a face model of 68 feature points has been released. However, since it is difficult to define contour points in the event image compared to the facial image of the frame-based camera, 32 facial feature points shown in this paper are defined in Figure 4.

This paper also investigates some Convolutional Neural Network (CNN) models using deep learning as a facial feature points detection method. Details will be described later in the experiment of Section 3.2.

### 2.4. Recognition model

The lipROI shown in Figure 3 is extracted based on the facial feature points detected above.

Temporal Convolutional Network (TCN) is a network that uses CNN for series data and has higher accuracy than RNN, such as LSTM, in tasks for time series data such as natural language and music (Bai et al., 2018). The TCN consists of a combination of a 1D fully-convolutional network and casual convolutions. Furthermore, Martinez et al. (2020) have proposed a model that uses the Multi-scale temporal convolutional network (MS-TCN). MS-TCN incorporates multiple time scales into the network to confuse short-term information with long-term information during feature coding. In this paper, MS-TCN is used.

**Figure 5:**
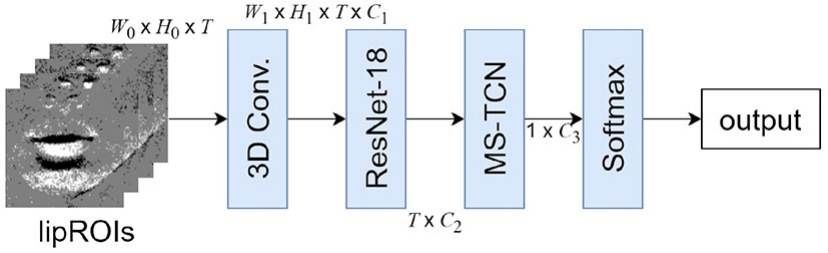
Model structure of recognition model.

The model structure used in this paper is shown in Figure 5. The model input is a time-series grayscale lipROIs, with the shape of *W*_0_ × *H*_0_ × *T*, where *T* stands for the temporal dimension and *W*_0_, *H*_0_ represent the width and height of the lipROIs, respectively. Next, the image features are extracted with ResNet-18, in which the first convolution layer is changed to 3D convolution to obtain the spatial-temporal features with shape *W*_1_ × *H*_1_ × *T* × *C*_1_, where *C*_1_ is the feature channel number. ResNet-18 is applied to produce features with shape *T* × *C*_2_. After that, it is composed of MS-TCN and Softmax through the Global average pooling layer. Here, *C*_1_, *C*_2_, and *C*_3_ denote different channel numbers. This structure is often used in lip-reading methods using frame image (Martinez et al., 2020). In the utterance scene, no large movements are observed around the lips. Therefore, ResNet-18, which is not a model with a complicated structure, can extract sufficient features.

### 2.5. Decision-level fusion

In this research, we acquire two modalities of frame and event images. Multimodal fusion is the process of integrating information from multiple sources for classification or regression tasks. There have been three information fusion approaches: early, hybrid, and late. Multimodal fusion provides the benefits of robustness, complementary information gain, and functional continuity of the system, even in the failure of one or more modalities. Early fusion integrates low-level features from each modality by correlation, potentially accomplishing the better task, but has difficulty in temporal synchronization among various input sources. Hybrid fusion attempts to exploit the advantages of both early and late fusion in a common framework. The late fusion obtains unimodal decision values and integrates them to obtain the final decision. Although the late fusion ignores low-level interactions between modalities, it allows easy training with more flexibility and simplicity to make predictions when one or more modalities are missing. In this research, we exploit late fusion.

## 3. Experiment

### 3.1. Face detection

**Figure 6:**
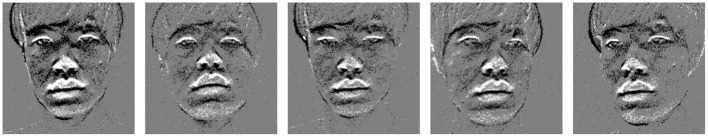
Face images taken in five directions (front, up, bottom, right, left).

As an experiment to verify the possibility of face extraction for event images, a nodding scene was originally taken with the cooperation of 23 students (16 males and seven females). Here, due to the robustness of the facial orientation, the subject shoots in five directions, front, top, bottom, left, and right, as shown in Figure 6. This five-direction shooting is defined as one set. For the four eyeglass wearers, the scene without eyeglasses was also taken. Those who did not wear eyeglasses shot three sets of 15 scenes per subject. As mentioned above in Section 2.2, one black image was taken out from each scene, and 15 face images were collected per subject. We collected 405 facial images from all 23 subjects.

If the subject wearing/not wearing eyeglasses is regarded as another subject, there are 27 subjects. We divided 27 people into three groups, with nine subjects in one group, and evaluated the face extraction accuracy by 3-fold cross-validation using two groups as training data and the remaining group as test data. The number of male and female eyeglass wearers in each group was set equally.

In this experiment, we trained a face detection model FDM_*e*_ on event images As a comparison method, we used the trained model FDM_*f*_ for the frame image provided in dlib. The success or failure of extraction was visually evaluated. The face detection success accuracy of the two models, FDM_*e*_ and FDM_*f*_, were 99.5 and 82.5%, respectively. From this, it was found that the face can be detected even in images taken with an event-based camera.

**Figure 7:**
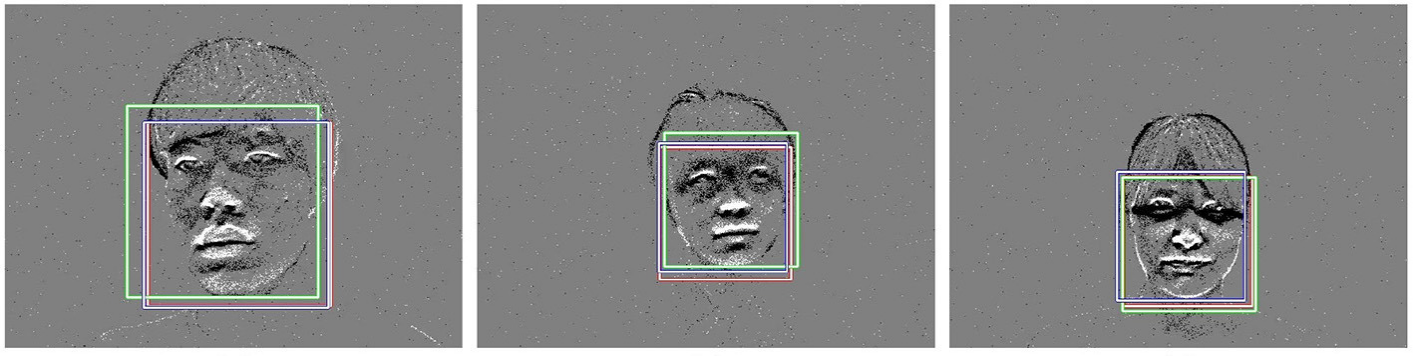
Face detection results (red: ground truth, green: model by frame-based image, blue: model by event image).

Figure 7 shows three examples of face detection results. In the figure, the red rectangle is the correct face rectangle, the green rectangle is the detection result of the model trained by the frame-based image, and the blue rectangle is the detection result of the model trained by the event image. In order to quantitatively evaluate the face detection results of Figure 7, the Intersection over Union (IoU) was calculated. Here, let IoU_*e*_ be the IoU between the detection result of the model trained by the event image and ground truth, and IoU_*f*_ be the IoU between the detection result of the model trained by the frame image and ground truth. IoU_*e*_ and IoU_*f*_ in the left image of Figure 7 are 0.955 and 0.737, respectively. IoU_*e*_ and IoU_*f*_ in the middle image of Figure 7 are 0.877 and 0.752, respectively. IoU_*e*_ and IoU_*f*_ in the right image of Figure 7 are 0.831 and 0.927, respectively. The left and middle images of Figure 7 are higher for IoU_*e*_. On the other hand, in the right image of Figure 7, IoU_*f*_ is higher than IoU_*e*_, but the difference between the two is small, and a sufficient result is obtained.

### 3.2. Facial feature points detection

In order to train and quantitatively evaluate feature point detection accuracy, one author manually gave the correct coordinates of all feature points to the 405 facial images collected in the previous experiment.

AlexNet (Krizhevsky et al., 2012), VGG16 (Simonyan and Zisserman, 2014), ResNet-50 (He et al., 2016), and ResNet-101 (He et al., 2016) were applied as models using deep learning. For the three models except for AlexNet, the trained model by ImageNet was used. As with face detection, we used a 68-points model (denoted as Kazemi68) provided by dlib as a trained model for frame-based images. On the other hand, the model trained at the 32 points shown in Figure 4 using the event-based image is denoted as Kazemi32. A normalized mean error (NME) was used to evaluate the feature point detection accuracy.

The experimental results are shown in Table 1. It was confirmed that the accuracy of the four types of CNN models increases as the number of layers increases. However, Kazemi32, which is non-deep learning, obtained the highest detection accuracy. The feature points are given to the boundaries of the parts of the face. The frame image has gradual shading changes, but the event image has many edges. Kazemi uses shape difference, which is suitable for images with many edges. Therefore, Kazemi has higher accuracy than CNN. In addition, Kazemi is a fast method without using deep learning. Therefore, Kazemi32 is used in subsequent recognition experiments.

**Table 1:** Feature points detection errors with various models.

**Model**	**NME**
Kazemi68	0.1413
Kazemi32	**0.0330**
AlexNet (Krizhevsky et al., 2012)	0.1233
VGG16 (Simonyan and Zisserman, 2014)	0.0422
ResNet50 (He et al., 2016)	0.0410
ResNet101 (He et al., 2016)	0.0372

Highest NME is highlighted in bold.

### 3.3. Isolated single sound recognition

This paper aims to estimate a single isolated sound using event images. However, there is no open dataset. Therefore, we originally collected utterance scenes with the cooperation of 20 students (13 males and seven females) and took the utterance scene. Here, the students in this experiment are different students from Section 3.1. The utterance contents are 15 Japanese sounds of /a/, /ka/, /sa/, /ta/, /na/, /ha/, /ma/, /ya/, /ra/, /wa/, /ga/, /za/, /da/, /ba/, and /pa/. These 15 sounds are conscious of consonant recognition, which is generally difficult to identify. The speaker was asked to utter 15 sounds one by one in one shooting. At this time, the speaker had his/her lips closed before and after the utterance. The interval between the sounds was set to 3 s. In the 15-sound utterance after the nodding motion, we instructed the subject not to move his head as much as possible. Each speaker was taken five times. In other words, we collected utterance scenes of 15 sounds × 5 times = 75 sounds per speaker.

Face detection and facial feature points detection were applied to the first nodding motion in each utterance scene, and the lipROI was obtained. Regarding the subsequent utterances of the 15 sounds, the same position as the lipROI obtained by the nodding motion was cropped. The utterance section was detected for the extracted lipROI.

**Figure 8:**
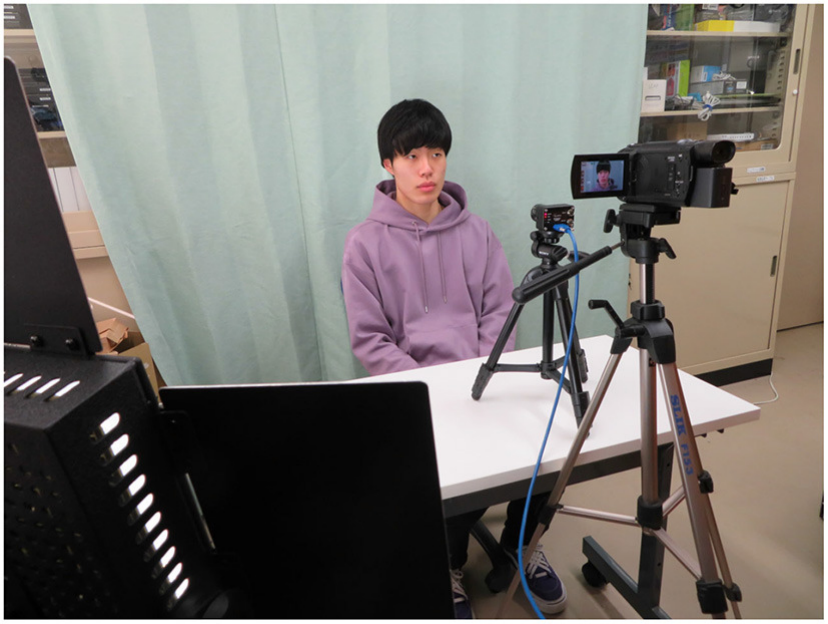
Utterance scene shooting environment.

In this experiment, DVXplorer by iniVation was used as the event camera. In addition, a video was shot simultaneously for comparison by a standard video camera. The shooting environment of the utterance scene is shown in Figure 8. Since the event and video cameras are independent devices, they cannot be installed on the same optical axis. Therefore, the event camera was placed in the front, the video camera in the rear, and each was fixed to a tripod, as shown in Figure 8. The speaker was seated on a chair and uttered in a front-facing position. The distance between the event camera and the subject was about 60cm. In order to shoot a bright face with a video camera, we put an LED light on it. A light green curtain was placed behind the speaker.

In the recognition experiment, 20 subjects were divided into four groups, and evaluation was performed for 4-fold cross-validation using 15 subjects in 3 groups as training data and five subjects in the remaining 1 group as test data. Here, the groups were created so that the male-female ratio was almost the same. We generated four frame rates for event images: 29.97, 60, 100, and 200 fps. The size of lipROI was 70 × 70 pixels. The model parameters were set to *W*_1_ = *H*_1_ = 15, *C*_1_ = 64, *C*_2_ = 512, and *C*_3_ = 768. The recognition results are shown in Table 2. The accuracy of the frame-based image is low, while the accuracy of the event image is high at all frame rates. With an event image, the higher the frame rate, the smaller movements can be captured, but the number of events per image decreases, and the number of information decreases. Therefore, it is assumed that the recognition accuracy decreased in event@100 and event@200. Lowering the frame rate increases the amount of information, but it is difficult to capture fine movements. There is a trade-off relationship between motion capture and the amount of information. Here, the highest recognition accuracy of 0.528 was obtained at 60 fps. Humans find the 15 sounds to be recognized difficult to identify, even visually.

**Table 2:** Isolated single sound recognition result.

**Image type**	**Frame rate [fps]**	**Accuracy**
Frame-based	29.97	0.290
Event	29.97	0.414
	60	**0.528**
	100	0.409
	200	0.399

Highest accuracy is highlighted in bold.

**Figure 9:**
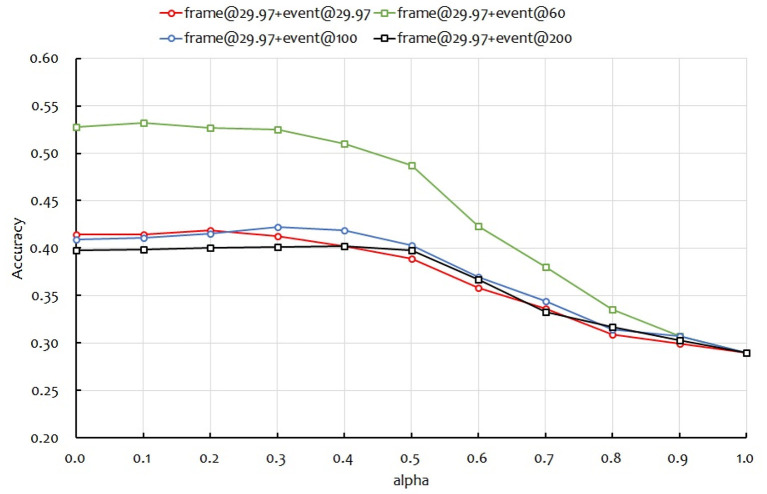
Recognition accuracies against α.

Next, we applied late fusion using two modalities: frame images and event images. The results are shown in Figure 9. In the figure, the vertical axis is the accuracy. α on the horizontal axis is the weight of the frame image and the event image, α = 0 is the accuracy when only the event image is used, and α = 1 is the accuracy when only the frame image is used. The event image used four frame rates in previous experiments: 29.97, 60, 100, and 200 fps. Therefore, Figure 9 also shows four curves. From Figure 9, the α values for the highest accuracy in multimodality fusion were 0.2, 0.1, 0.3, and 0.4 for event images of 29.97, 60, 100, and 200 fps, respectively. There was a strong negative correlation between the recognition accuracy and α in a single modality using only event images. When α = 0.1 and the event image is 60 fps, we obtained a recognition accuracy of 0.532, which is higher than using one modality. We confirmed that the two modalities improve accuracy, albeit only slightly. Here, we applied early fusion after ResNet-18 in Figure 1 to combine the features of the two modalities, frame@29.97 and event@29.97, and recognize them with MS-TCN. As a result, the recognition rate was 0.400, which was lower than the single modality. Thus, it was confirmed the effectiveness of the proposed late fusion approach.

**Figure 10:**
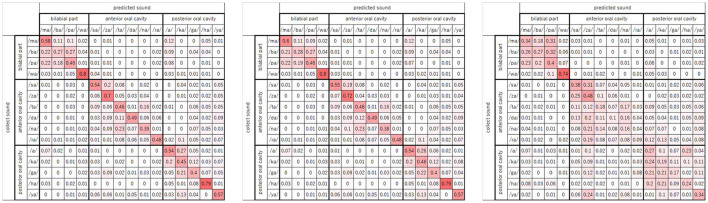
Consugion matrices (**left**: α = 0.0, **center**: α = 0.1, **right**: α = 1.0).

Finally, Figure 10 shows three confusion matrice of frame@29.97 (Figure 10, right) and event@60 (Figure 10, left) and two modalities of frame@29.97 and event@60 (Figure 10, center). The numerical value of each cell is the recognition accuracy, and cells with higher recognition accuracy are drawn in red. Here, the arrangement of the 15 sounds was divided into three articulation groups, the bilabial part (four sounds: /ma/, /ba/, /pa/, and /wa/), the anterior part of the oral cavity (six sounds: /sa/, /za/, /ta/, /da/, /na/, and /ra/), and the posterior part of the oral cavity (five sounds: /a/, /ka/, /ga/, /ha/, and /ya/), according to the place of articulation. The place of articulation is in the oral cavity, where the consonant is pronounced. It is considered to be related to the expression of the mouth shape. For α = 0.0 and α = 0.1, wrong recognitions tend to be in the articulation group sound. From these matrices, it can be found that the event image captures the movement when the consonant is uttered.

## 4. Conclusion

This paper proposed a lip-reading method using an event-based camera and conducted a recognition experiment for 15 Japanese sounds. As a result, we confirmed that event images have higher recognition accuracy than traditional frame-based images. We also showed that the face detection and facial feature point detection methods proposed for the frame-based image are also applicable to the event image.

In this paper, we focused on the isolated single sound that is difficult to recognize, but we will develop them into words and sentences in the future.

## Data availability statement

The datasets presented in this article are not readily available because our dataset contains facial images and can identify individuals. Requests to access the datasets should be directed to TS, saitoh@ai.kyutech.ac.jp.

## Ethics statement

Written informed consent was obtained from the individual(s) for the publication of any potentially identifiable images or data included in this article.

## Author contributions

TK and TS contributed to the conception and design of this study. TA created the MS-TCN model. All authors contributed to the revision of the manuscript, read, and approved the submitted version.

## Footnotes

^1^ http://dlib.net/

^2^ https://google.github.io/mediapipe/solutions/face_detection
